# Clinical Outcomes Associated with a Propolis-Based Nano-Formulated Bioadhesive Oral Gel: A Retrospective Case Series and Non-Randomized Controlled Study in Patients with Intellectual Disabilities

**DOI:** 10.3390/gels12060490

**Published:** 2026-06-02

**Authors:** György Szmirnov, Ilona Szmirnova, Ákos Tamás Nagy, Gábor Kammerhofer, Zsolt Németh, Zsófia Zubor, György Szabó

**Affiliations:** Department of Oro-Maxillofacial Surgery and Stomatology, Semmelweis University, 1085 Budapest, Hungary; szmirnov.gyorgy@semmelweis.hu (G.S.); szmirnova.ilona@semmelweis.hu (I.S.); nagy.akos02@stud.semmelweis.hu (Á.T.N.); kammerhofer.gabor@semmelweis.hu (G.K.); nemeth.zsolt@semmelweis.hu (Z.N.); zubor.zsofia@stud.semmelweis.hu (Z.Z.)

**Keywords:** propolis, vitamin C, vitamin E, oral mucosa, intellectual disabilities, xerostomia, oral mucositis

## Abstract

A propolis-based nano-formulated bioadhesive oral gel (NBF gel) containing vitamins C and E has been proposed as a supportive topical therapy for oral mucosal lesions. The aim of this study was to evaluate clinical outcomes associated with the use of the gel during a 15-year institutional clinical experience and to assess its adjunctive effect on periodontal status in patients with intellectual disabilities. The study consisted of two components: a retrospective observational case series and a non-randomized controlled clinical study. In the retrospective component, 295 patients (219 females and 76 males) received topical NBF gel treatment for various oral mucosal conditions, including xerostomia-associated mucositis, inflammatory lesions, aphthous ulcers, herpes infections, glossodynia, leukoplakia, erythroplakia, and post-surgical conditions. Treatment response was assessed descriptively using patient-reported symptom improvement combined with clinical evaluation. Overall, treatment was considered successful in 265/295 patients (89.8%), while 14/295 patients (4.7%) were classified as ineffective and 16/295 patients (5.4%) as inconclusive. More favorable responses were observed in inflammatory and post-treatment lesions than in potentially premalignant or neuropathic conditions. In the controlled periodontal component, 40 patients with mild to moderate intellectual disabilities were allocated into a control group performing toothbrushing alone and a test group additionally receiving topical NBF gel application. Periodontal status was assessed using the Basic Periodontal Examination (BPE) index at baseline and after 1 and 2 weeks. Adjunctive gel application was associated with greater improvement in periodontal status compared with toothbrushing alone. No clinically relevant adverse effects were documented during the observation period; however, because adverse events were not assessed using a predefined safety-monitoring protocol, these findings should be interpreted cautiously. The present findings suggest that the investigated nano-formulated bioadhesive oral gel may represent a potentially useful adjunctive topical therapy in selected oral mucosal and periodontal conditions. Further randomized controlled studies with standardized objective outcome measures are required to confirm these preliminary findings.

## 1. Introduction

Nano-formulated bioadhesive gels have emerged as potential adjunctive approaches in the management of oral mucosal lesions. In recent years, increasing attention has been directed toward bioadhesive drug delivery systems in oral medicine because these formulations may prolong the contact time of active substances with the mucosal surface, thereby potentially enhancing local bioavailability. This may be particularly relevant in the oral cavity, where salivary flow and mechanical forces can reduce the effectiveness of conventional topical agents. Consequently, nano-formulated bioadhesive gels have attracted interest as possible supportive therapies in oral mucosal management.

Among these, a propolis-based gingival gel containing vitamins C and E has gained attention because of its potential bioadhesive and antioxidant properties [1,2,3,4,5,6,7,8,9,10,11,12,13,14]. The nano-emulsion formulation may contribute to prolonged local retention on the mucosal surface, while vitamins C and E are well-established antioxidants that may support tissue repair processes and reduction in oxidative stress [4,15,16,17]. Propolis, a natural bee-derived product, has been reported to exhibit antibacterial, antifungal, and anti-inflammatory properties and may contribute to mucosal protection and healing [15,16,17,18,19,20]. However, the exact molecular and microbiological mechanisms associated with the formulation were not directly investigated in the present study.

Patients with intellectual disabilities represent a particularly vulnerable group with regard to oral health. Previous studies conducted by our group demonstrated that their oral health status is significantly worse compared to both the general population and individuals with other forms of disability [21,22]. Based on these findings, a preventive oral hygiene protocol was developed to improve periodontal outcomes in this population. The present study represents a continuation of this preventive approach. The study had two primary objectives. First, to summarize retrospective clinical outcomes associated with the use of the investigated gel in various oral mucosal conditions over a 15-year institutional clinical experience. Second, to evaluate its effect on periodontal status in a non-randomized controlled study involving patients with intellectual disabilities.

## 2. Results and Discussion

### 2.1. Retrospective Clinical Outcomes

**Figure 1:**
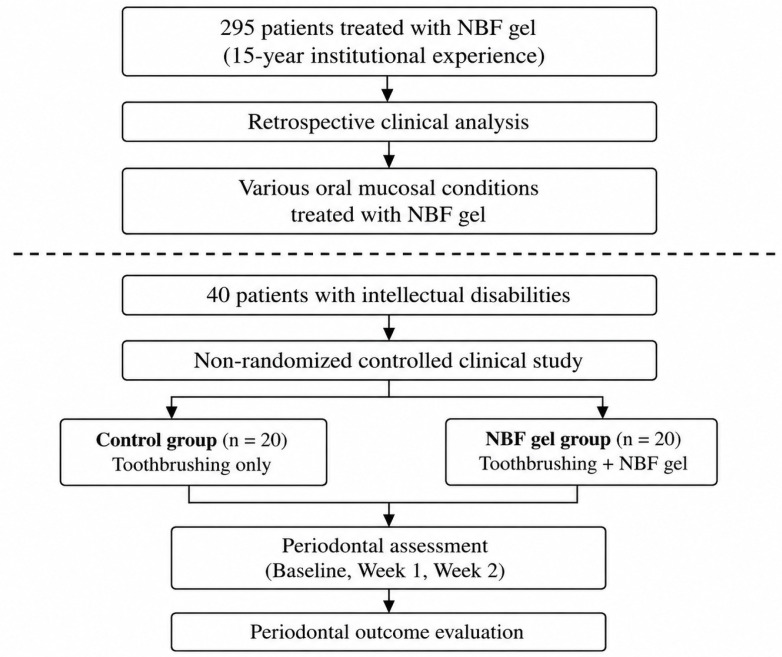
Study design and workflow of the retrospective observational case series and the non-randomized controlled clinical study. The dashed line indicates the distinction between the retrospective study and the non-randomized controlled clinical study.

The retrospective analysis included 295 patients treated with NBF gel for various oral mucosal conditions. The study population consisted of 219 females (74.2%) and 76 males (25.8%). The overall study design and workflow are illustrated in Figure 1.

Clinical outcomes according to diagnostic category are summarized in Table 1. The highest response rates were observed in patients with xerostomia-associated mucositis, in whom clinical improvement was reported in all 165 cases. Similarly, favorable clinical outcomes were observed following intraoral laser surgery (11/11 cases) and post-surgical application of the gel (35/40 cases). Improvement was also observed in most patients with inflammatory oral mucosal conditions of various etiologies (30/35 cases) and in patients with aphthous ulcers or herpes infections (15/17 cases). In contrast, treatment response appeared to be more limited in leukoplakia, erythroplakia, and glossodynia. No clinically relevant improvement was observed in leukoplakia, while partial responsiveness was observed in erythroplakia (3/9 cases). In glossodynia, improvement was observed in 6 out of 12 patients.

Overall, treatment was considered successful in 265/295 patients (89.8%), ineffective in 14/295 patients (4.7%), and inconclusive in 16/295 patients (5.4%). No clinically relevant adverse effects associated with topical NBF gel application were documented in the available clinical records during the retrospective observational period. Because the retrospective cohort included heterogeneous oral mucosal conditions with different clinical characteristics and natural histories, these findings should be interpreted descriptively and considered exploratory.

### 2.2. Periodontal Outcomes in Patients with Intellectual Disabilities

**Figure 2:**
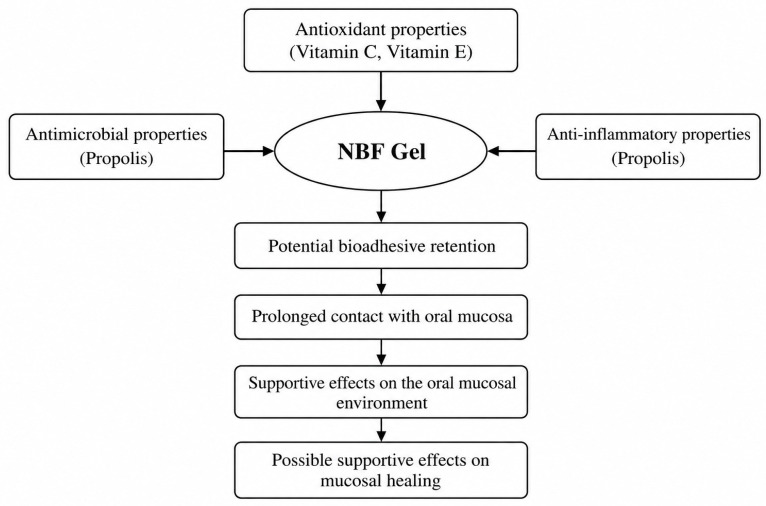
Proposed literature-based mechanisms potentially associated with the clinical effects of the investigated NBF gel, including antioxidant, anti-inflammatory, and antimicrobial properties, prolonged mucosal retention, and supportive effects on oral mucosal healing. The proposed mechanisms were not directly investigated in the present study.

No clinically relevant adverse effects associated with the topical application of the gel were reported during the controlled study period. Because only aggregated group-level periodontal data were available for analysis and the study was non-randomized, the findings of the controlled clinical component should be interpreted descriptively and considered exploratory. Proposed literature-based mechanisms potentially associated with the observed clinical findings are summarized in Figure 2.

**Figure 3:**
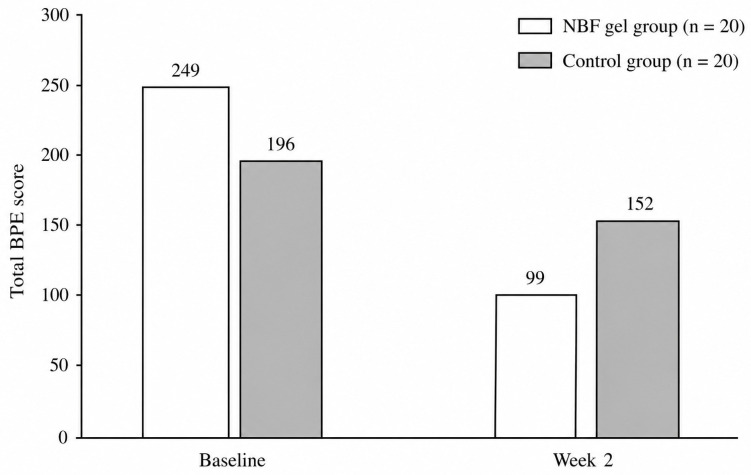
Descriptive changes in total BPE scores between baseline and week 2 in the NBF gel and control groups. Both groups demonstrated improvement in periodontal status during the observation period; however, a greater reduction in total BPE scores was observed in the group receiving adjunctive NBF gel treatment. Because only aggregated group-level data were available, variability measures and statistical significance indicators are not presented.

In the gel-treated group, the total BPE score decreased from 249 at baseline to 99 at the final assessment, corresponding to a 60.2% reduction. In the control group, the total BPE score decreased from 196 to 152, corresponding to a 22.4% reduction. Changes in periodontal status during the observation period are summarized in Table 2. From baseline to week 2, the relative improvement was 60.2% in the NBF gel group and 22.4% in the control group. A greater reduction in BPE scores was observed in the NBF gel group throughout the observation period. Figure 3 illustrates the temporal changes in periodontal status during the study period. Both groups demonstrated gradual improvement over time; however, the reduction in BPE scores appeared to be more pronounced in patients receiving adjunctive NBF gel treatment.

Periodontal outcomes in the controlled clinical study are summarized in Table 2 and Figure 3. Improvement in periodontal status was observed in both groups during the observation period. However, the magnitude of improvement was greater in the group receiving adjunctive NBF gel treatment.

### 2.3. Clinical Implications and Therapeutic Perspectives

The present study provides retrospective observational data and preliminary non-randomized controlled findings that may suggest a potential adjunctive role for a propolis-based nano-formulated bioadhesive oral gel in selected oral mucosal and periodontal conditions [1,2,3,4,5,6,7,8,9,10,11,12,13,14]. The retrospective component demonstrated favorable clinical responses in several inflammatory and post-treatment oral mucosal conditions, whereas the controlled periodontal component suggested greater improvement in periodontal status when the gel was used in combination with routine oral hygiene measures.

Patients with intellectual disabilities represent a particularly vulnerable population with regard to oral health [4,21,22]. Previous investigations have demonstrated poorer oral hygiene and higher prevalence of periodontal disease in this population, frequently associated with impaired manual dexterity, dependence on caregivers, and limited access to regular dental care [23,24,25]. In this context, supportive topical therapies that can be incorporated into routine oral hygiene procedures may have practical clinical relevance. The ease of application and local mode of action of the investigated formulation may support patient adherence and allow integration into daily oral care protocols without substantial additional burden.

In the retrospective observational cohort, the highest response rates were observed in patients with xerostomia-associated mucositis and in post-surgical conditions. These findings may reflect the importance of local mucosal protection and prolonged retention of topical formulations in situations where mucosal integrity is compromised [6,7,23,24,26,27,28,29]. In contrast, more limited clinical responsiveness was observed in leukoplakia, erythroplakia, and glossodynia. These differences may indicate that conditions with predominantly inflammatory or reversible mucosal alterations respond more favorably to supportive topical therapy than lesions associated with structural, neuropathic, or potentially premalignant changes. Because the retrospective cohort included heterogeneous diseases with substantially different biological behavior and clinical course, the observed response rates should be interpreted descriptively and considered exploratory. The controlled clinical study involving patients with intellectual disabilities demonstrated improvement in periodontal status in both study groups during the observation period. However, a greater reduction in total BPE scores was observed in patients receiving adjunctive NBF gel treatment in addition to routine toothbrushing. Although the study design does not allow causal conclusions, these findings may suggest that topical bioadhesive formulations could provide additional benefit when combined with conventional oral hygiene measures. Similar observations have previously been reported in studies evaluating bioadhesive oral delivery systems designed to prolong local retention of active substances within the oral cavity [2,5,9,30,31,32,33,34]. The potential biological mechanisms underlying the observed clinical effects were not directly investigated in the present study. However, previous experimental and clinical reports suggest that propolis possesses antibacterial, antifungal, antioxidant, and anti-inflammatory properties that may contribute to reduction in local inflammation and support mucosal healing [15,18,19,20]. Vitamins C and E are well-established antioxidants that may contribute to tissue repair processes and reduction in oxidative stress [4,16,17]. In addition, the nano-formulated bioadhesive structure of the investigated gel may facilitate prolonged mucosal retention and sustained local exposure to active substances [4,6,7,19,26,32]. Nevertheless, these proposed mechanisms remain literature-based assumptions and should be interpreted cautiously.

Several limitations of the present study should be acknowledged. First, the retrospective observational design limits standardization of data collection and introduces potential selection bias. Second, the retrospective cohort included highly heterogeneous oral mucosal conditions with different etiologies, biological behavior, and expected healing patterns, which limits direct comparability between subgroups. Third, treatment response assessment relied primarily on subjective symptom improvement combined with routine clinical evaluation, and no validated objective scoring system was consistently available during the retrospective observation period. Fourth, the controlled periodontal study was non-randomized, unblinded, and conducted on a relatively small sample size over a short follow-up period. In addition, only aggregated group-level periodontal data were available for analysis, and baseline differences in BPE scores between the groups may have influenced the magnitude of observed improvement. The study also lacked placebo control, formal sample-size calculation, standardized adverse-event monitoring, and mechanistic laboratory investigations. Consequently, the findings should be interpreted cautiously and considered preliminary. No clinically relevant adverse effects associated with topical application of the gel were documented in the available clinical records or reported during follow-up visits. However, because adverse events were not assessed using a predefined safety-monitoring protocol, the safety findings remain limited. This consideration may be particularly relevant because propolis-containing products have the potential to induce hypersensitivity reactions in susceptible individuals [15,18].

Future research should focus on larger randomized controlled trials with standardized objective outcome measures, longer follow-up periods, and blinded study design whenever feasible. Additional studies evaluating microbiological, inflammatory, and oxidative stress parameters may help clarify the biological mechanisms associated with the investigated formulation. Further investigation into optimal application frequency, treatment duration, and potential use in oral surgical wound healing may also be clinically valuable. Because impaired intraoral wound healing may occur in systemic conditions such as diabetes mellitus and medication-related osteonecrosis of the jaw (MRONJ), supportive topical therapies aimed at improving mucosal healing may warrant additional investigation in these clinical settings [35,36,37,38,39,40,41].

## 3. Conclusions

The findings of the present study suggest that the investigated propolis-based nano-formulated bioadhesive oral gel may have potential as an adjunctive topical therapy in selected oral mucosal and periodontal conditions. Favorable clinical outcomes were observed particularly in inflammatory and post-surgical oral mucosal lesions, whereas more limited responsiveness was observed in leukoplakia, erythroplakia, and glossodynia. In the controlled clinical component, adjunctive application of the gel was associated with greater improvement in periodontal status compared with routine oral hygiene measures alone in patients with intellectual disabilities. However, because of the retrospective observational design, heterogeneity of the included clinical conditions, non-randomized study design, short follow-up period, and limited sample size, the findings should be interpreted cautiously and considered preliminary. Further well-designed randomized controlled trials with standardized objective outcome measures are required to clarify the clinical applicability and therapeutic potential of such bioadhesive formulations in oral soft tissue therapy.

## 4. Materials and Methods

### 4.1. Ethics Approval and Study Design

This study was conducted in accordance with the Declaration of Helsinki and was approved by the Regional and Institutional Committee of Science and Research Ethics of Semmelweis University (approval number: SE RKEB 68/2025, approval date: 30 April 2025). For the retrospective component, anonymized clinical records collected during routine patient care were analyzed retrospectively following ethics committee approval. For the controlled clinical study, participants with mild to moderate intellectual disabilities were recruited from a specialized institutional care facility. Written informed consent for participation and publication of anonymized clinical data was obtained from the participants whenever possible, as well as from their legal guardians or authorized representatives prior to inclusion in the study.

The study consisted of two components: (1) a retrospective observational case series involving patients treated with the investigated gel for various oral mucosal conditions over a 15-year institutional clinical period; and (2) a non-randomized controlled clinical study evaluating periodontal outcomes in patients with intellectual disabilities.

### 4.2. Retrospective Clinical Analysis

Over a 15-year period, a total of 295 patients were treated with NBF gel for various oral mucosal conditions (Table 1). The cohort included 219 females and 76 males. Patients were included if they had a documented clinical diagnosis of an oral mucosal condition, received NBF gel according to the institutional treatment protocol, and attended at least one follow-up visit within the defined assessment period. Patients were excluded if treatment records were incomplete, the diagnosis was unclear, or treatment outcome could not be reliably determined from the clinical documentation. Among these patients, 165 presented with xerostomia-associated mucositis. The underlying causes of xerostomia included radiotherapy, chemotherapy, medication-related side effects, hormonal imbalance, and Sjögren’s syndrome.

Additional clinical conditions included glossodynia (*n* = 12), aphthous ulcers and herpes infections (*n* = 17), inflammatory conditions of various etiologies (*n* = 35), leukoplakia (*n* = 6), erythroplakia (*n* = 9), and post-laser surgical conditions (*n* = 11). Furthermore, the gel was applied in 40 cases immediately following oral surgical procedures. Because the retrospective cohort included heterogeneous oral mucosal conditions with substantially different biological characteristics and clinical behavior, treatment outcomes were interpreted descriptively and considered exploratory in nature. The subgroup findings suggest that inflammatory and post-treatment lesions may be more responsive than potentially premalignant or neuropathic conditions.

### 4.3. Investigated Formulation

The investigated product was a commercially available propolis-based nano-formulated bioadhesive oral gel (NBF Gingival Gel, Sungwon Pharmaceutical Co., Ltd., Paju-si, Gyeonggi-do, Republic of Korea, registered No. 0763). According to the manufacturer, the formulation contains propolis extract, vitamins C and E (ascorbic acid and tocopheryl acetate), xylitol, menthol, peppermint oil, silica, glycerin, cellulose gum, sodium monofluorophosphate, PEG-1500, and additional excipients intended to support local bioadhesion and oral application.

The product was used as part of routine clinical care according to the manufacturer’s instructions. The gel was not experimentally modified or reformulated by the authors. The manufacturer had no role in study design, data collection, statistical analysis, manuscript preparation, or publication decisions. The product was obtained through routine commercial availability, and no financial support was received from the manufacturer. The physicochemical characteristics of the nano-formulation were not independently analyzed in the present study and were based on manufacturer-provided information.

### 4.4. Treatment Protocol

Patients were instructed to apply the NBF gel twice daily following toothbrushing in the morning and evening. The gel was spread onto the oral mucosa, particularly over the affected mucosal surfaces, using a clean finger. Patients were instructed to avoid eating or drinking for 30 min following application in order to prolong local mucosal retention of the formulation. Concurrent medications and routine therapies were continued as prescribed during the observational period. In the controlled periodontal study, participants in the test group additionally applied the gel 2–3 times daily in combination with routine toothbrushing procedures.

### 4.5. Outcome Assessment

Treatment effectiveness was assessed using a subjective symptom-improvement scale ranging from 1 to 10 (1 = no improvement; 10 = complete symptom resolution), in combination with clinical evaluation of mucosal healing. Outcomes were categorized as follows. Successful outcomes were defined as a symptom-improvement score ≥ 7 combined with visible clinical improvement or mucosal healing. Ineffective outcomes were defined as a symptom-improvement score ≤ 3 without observable clinical improvement. Inconclusive outcomes were defined as symptom-improvement scores between 4 and 6, inconsistent clinical findings, incomplete follow-up, or uncertain relationship between treatment and clinical response.

Clinical evaluation was performed approximately one week after treatment initiation. Because no validated symptom-scoring system was routinely used during the retrospective observational period, treatment response assessment should be interpreted descriptively. Objective measurement of salivary flow was not performed, as the gel is not intended to increase saliva production. All clinical evaluations were performed by the same examiner to reduce inter-examiner variability. No adverse effects were documented in the available clinical records or reported during follow-up visits. However, because adverse events were not assessed using a predefined safety-monitoring protocol, these findings should be interpreted cautiously.

### 4.6. Controlled Study in Patients with Intellectual Disabilities

A non-randomized controlled clinical study was conducted in 40 patients with mild to moderate intellectual disabilities who had previously received instruction in proper toothbrushing techniques. Participants were recruited from a specialized institutional care facility for individuals with intellectual disabilities. All eligible participants attending the Department during the study period were included. Participants from other institutions were not enrolled. Participants were allocated into two groups: a control group (*n* = 20) performing toothbrushing alone and a test group (*n* = 20) receiving adjunctive topical NBF gel application in addition to toothbrushing.

Formal randomization and blinding were not performed. Patients were followed for 2 weeks after baseline, with periodontal assessments performed at baseline, week 1, and week 2. Periodontal status was evaluated using the BPE index with a WHO (World Health Organization) periodontal probe. Each tooth in all six sextants was examined. BPE scores ranged from 0 (healthy periodontal condition) to 4 (severe periodontal involvement). For each sextant, the highest recorded BPE score was registered, and the sum of sextant scores was used to characterize overall periodontal status. All periodontal examinations were performed by the same clinician to minimize inter-examiner variability.

### 4.7. Statistical Analysis

Statistical analysis was performed primarily for the controlled periodontal study. Due to the retrospective and heterogeneous nature of the retrospective clinical dataset, descriptive statistics were considered the most appropriate analytical approach for that component. Continuous variables, where applicable, are presented as mean ± standard deviation (SD), while categorical variables are expressed as frequencies and percentages. Because BPE scores represent ordinal repeated-measure data and the study population was limited in size, nonparametric statistical methods were considered the most appropriate analytical approach for exploratory comparison of periodontal outcomes. However, because only aggregated group-level periodontal data were available for the final retrospective analysis, the periodontal findings are presented descriptively. Relative changes in total summed BPE scores between baseline and week 2 were therefore reported without formal inferential statistical testing. Due to the exploratory design, absence of randomization, and limited sample size, the findings should be interpreted cautiously. Statistical analyses were performed using IBM SPSS Statistics software (version 29; IBM Corp., Armonk, NY, USA).

## Figures and Tables

**Table 1:** Distribution of oral mucosal conditions included in the retrospective observational cohort treated with topical NBF gel.

Condition	Number of Patients (*n*)
Xerostomia-associated mucositis	165
Glossodynia	12
Aphthous ulcers and herpes infections	17
Inflammatory conditions (other etiologies)	35
Leukoplakia	6
Erythroplakia	9
Post-laser surgery	11
Post-surgical application	40
Total	295

**Table 2:** Changes in periodontal status during the controlled clinical study. Relative improvement was calculated based on the change in total summed BPE scores between baseline and week 2.

Group	Baseline Total BPE Score	Final Total BPE Score	Relative Improvement (%)
NBF gel group	249	99	60.2%
Control group	196	152	22.4%

## Data Availability

The data presented in this study are available from the corresponding author upon reasonable request.

## Abbreviations

BPE	basic periodontal examination
MRONJ	medication-related osteonecrosis of the jaw
NBF	propolis-based nano-formulated bioadhesive oral gel
SD	standard deviation
WHO	world health organization
